# Evaluation of Systemic Inflammation Before and After Standard Anti-tuberculosis Treatment in Patients With Active Pulmonary Tuberculosis and Diabetes Mellitus

**DOI:** 10.7759/cureus.55391

**Published:** 2024-03-02

**Authors:** Jesús Andrés López-González, Juan Manuel Martínez-Soto, Carolina Avila-Cervantes, Ana Lourdes Mata-Pineda, Gerardo Álvarez-Hernández, Jehan Bonizu Álvarez-Meza, Enrique Bolado-Martínez, Maria del Carmen Candia-Plata

**Affiliations:** 1 Department of Chemical-Biological Sciences, University of Sonora, Hermosillo, MEX; 2 Department of Medicine and Health Sciences, University of Sonora, Hermosillo, MEX; 3 Department of Pathogens Laboratory, State Public Health Laboratory, Hermosillo, MEX; 4 Department of Health Promotion and Disease Prevention, Sonora Health Services, Hermosillo, MEX

**Keywords:** sputum, inflammation, cytokines, diabetes, tuberculosis

## Abstract

Background

Diabetes mellitus (DM) is a common comorbidity of active pulmonary tuberculosis (APTB) that increases the risk of treatment failure during anti-tuberculosis chemotherapy. Evaluating systemic inflammatory response could help determine differences in response to treatment between APTB patients and those with APTB and DM.

Methodology

To explore changes in systemic inflammation, measured by a set of inflammatory mediators in subjects with APTB and TBDM before and after six months of anti-tuberculosis chemotherapy, 30 APTB and nine TBDM subjects underwent cytokine testing, including interleukin (IL)-6, IL-8, IL-10, interferon-gamma (IFN-γ), tumor necrosis factor-alpha (TNF-α), and transforming growth factor-beta 1 (TGF-β1) by enzyme-linked immunosorbent assay, C-reactive protein by nephelometry, and sialic acid by colorimetric assay at baseline and following six months of standard anti-tuberculosis treatment. Sputum smear microscopy or molecular biology (Xpert MTB/RIF) was used for diagnosis, and sputum smear microscopy was performed monthly during the treatment of the patient with pulmonary tuberculosis to evaluate his evolution. Principal component analysis examined changes in the inflammatory status.

Results

Both groups showed negative sputum smear microscopy in the sixth month after starting anti-tuberculosis chemotherapy. TGF-β1 was found to be significantly higher in subjects with TBDM before treatment compared to APTB patients (p<0.001), and systemic inflammation continued only in TBDM subjects after treatment (accumulation and persistence of inflammatory mediators like IL-6, IL-8, IL-10, IFN-γ, TNF-α, TGF-β1, C-reactive protein, and sialic acid in blood). On the other hand, the mediators IFN-γ, C-reactive protein, and total sialic acid were found to be most influential in distinguishing pre- and post-treatment inflammatory response in subjects with APTB without DM.

Conclusions

Inflammatory mediators analyzed in combination, including IFN-γ, CRP, and total sialic acid, may be useful in evaluating the systemic inflammatory response in subjects with APTB and TBDM before and after anti-tuberculosis treatment. Determining these mediators revealed persistent systemic inflammation in TBDM subjects after six months of standard tuberculosis treatment, despite negative sputum smear microscopy results and good glycemic control. This suggests a need for inflammation-modulating therapies during tuberculosis control. Finally, monitoring sputum smear microscopy results alongside the determination of proposed inflammatory mediators (IFN-γ, CRP, and total sialic acid) are effective in evaluating the response to anti-tuberculosis treatment in APTB subjects without DM, warranting further investigation.

## Introduction

Tuberculosis (TB) is the leading cause of death from an infectious disease worldwide, except for that caused by coronavirus disease 2019 (COVID-19) in 2022. An estimated global total of 10.6 million new cases of TB occurred in 2022 [1], with a higher risk in those with diabetes mellitus (DM), undernourishment, heavy alcohol consumption, and acquired immunodeficiency syndrome [2]. In the presence of DM, the risk of contracting TB escalates three-fold [3], and DM also increases TB treatment failure and TB relapse [4-6]. In addition, pathways connected to epigenetic reprogramming that are related to diabetic complications are amplified in patients with active pulmonary tuberculosis (APTB) and patients with APTB and DM (TBDM), compared to those with DM alone [7].

The mechanism underlying the increased susceptibility to the development of active tuberculosis in people with DM is still poorly understood. Experimentally, hyperglycemia has been observed to impair innate immune responses to infection, which may produce a defective innate response to *Mycobacterium tuberculosis* (Mtb), resulting in a critical delay of adaptive immunity [8]. This would partly explain the greater susceptibility of patients with DM to developing APTB, but studies continue to be carried out to identify the causal mechanisms and how DM affects the clinical course of patients with tuberculosis [9,10].

The primary treatment of a new case of APTB - defined as a patient in whom the diagnosis of pulmonary tuberculosis has been established for the first time or if treatment was received for less than 30 days - should include monthly follow-up with sputum smear microscopy until the end of treatment [11]. This follow-up is essential for the clinical control of the disease, and in adults, it is performed through microscopic examination of sputum [12,13]. While this test is simple, rapid, and low-cost, its low sensitivity limits its usefulness as a tool for monitoring the treatment outcome of patients with APTB [14,15].

In recent decades, methods for monitoring treatment response have emerged due to research on the systemic inflammatory process associated with mycobacterial infection. Several markers appear to have independent clinical value for both diagnosis and assessment of anti-TB treatment response, but their effectiveness remains inconclusive [16-18]. Little is known about the usefulness of acute-phase markers, such as C-reactive protein (CRP) or sialic acid [19-21] However, CRP may correlate with APTB treatment response, while sialic acid seems to differentiate between uninfected individuals, infected individuals, and patients with active TB [22]. It also correlates with other acute-phase reactants and remains stable during the acute-phase response [23,24].

It is important to highlight that both APTB and DM are diseases that cause systemic inflammation. The concurrence of these conditions has been linked to a greater burden of systemic inflammation, particularly in individuals with poorly controlled DM [25,26]. Studies evaluating the effects of hyperglycemia on cytokine production in vitro and during mycobacterial infection have shown that hyperglycemia alters cytokine production. Specifically, it increases levels of cytokines such as interleukin (IL)-1, IL-6, IL-10, and tumor necrosis factor-alpha (TNF-α) [27]. Furthermore, one study investigated the immune response of mice to Mtb infection after inducing type 2 DM. They observed significantly higher expression of genes encoding both pro- and anti-inflammatory cytokines compared to mice with DM alone or non-diabetic mice infected with Mtb [28].

Patients with TBDM exhibit high cytokine levels during anti-tuberculosis treatment. This may contribute to the inadequate clinical outcomes observed in patients with TBDM during anti-tuberculosis treatment [29]. Additionally, it has been suggested that patients with TBDM experience changes in the plasma cytokine network, resulting in a characteristic cytokine pattern. This pattern is thought to arise from epigenetic reprogramming and neutrophilic inflammation in these patients [7].

It is known that during the chronic inflammatory process that characterizes both APTB and DM, certain proinflammatory cytokines, such as IL-6 and TNF-α, induce the synthesis of acute-phase reactants [30,31]. For this reason, several studies have attempted to demonstrate that, in addition to inflammatory cytokines, acute-phase reactants could be used as markers of tuberculous activity [16-18,32]. If this is the case, the assessment of systemic inflammatory response by measuring both a series of cytokines and acute-phase reactants could be useful for evaluating the response to anti-TB treatment and monitoring the clinical management of patients with concurrent TB and DM.

In this study, we aimed to explore whether changes in systemic inflammation could be explained by a set of inflammatory mediators, including IL-6, IL-8, IL-10, interferon-gamma (IFN-γ), TNF-α, transforming growth factor beta 1 (TGF-β1), CRP, and total sialic acid, in subjects with APTB and those with TBDM before and after six months of standard TB treatment.

## Materials and methods

Study design, patients, and treatment

The present work is a prospective longitudinal study, which was approved by the Bioethics Research Committee of the Department of Medicine and Health Sciences at the University of Sonora (#DMCS/CBICMCS/D-119) (CEI NIH: #IORG0008079). It was conducted in eight primary health care units of the Sonora Health Services located in Hermosillo, Mexico. All patients were informed about the specifics of their involvement in the study and gave their written consent before enrollment.

Adults of both sexes aged 18 years or older, diagnosed with incident or relapsed APTB, were included in the study. Patients meeting the aforementioned criteria, as well as those with DM (HbA1c ≥6.5%) who completed the final study visit (month six), were sequentially selected for inclusion in the TBDM group. All patients received care from primary health care units under Sonora Health Services and were about to initiate the first phase of standard tuberculosis treatment. Additionally, they were registered in the Mexican National Epidemiological Surveillance Platform for Tuberculosis Cases of the National Epidemiological Surveillance System (SINAVE) (https://www.sinave.gob.mx/) [33]. Anthropometric data, clinical signs and symptoms, and results of sputum smear microscopy for each subject were obtained from the SINAVE platform. We verified this information by reviewing the clinical records of all subjects and contacted their physicians for clarification when discrepancies arose. Patients in both groups were excluded if they had conditions preventing blood sample collection, extrapulmonary tuberculosis, confirmed human immunodeficiency virus (HIV) infection, or any other infectious or non-infectious pathology (excluding DM) contributing to systemic inflammation, a typical, multisystemic, and phase-specific pathological process resulting from systemic damage [29,32]. Cases of patients who abandoned anti-TB treatment were also removed from the study.

Thirty patients diagnosed with APTB (incident or relapse) and nine patients with TBDM (incident or relapse) were recruited during the period from February 2019 to December 2020. All 39 patients were administered the treatment recommended by the World Health Organization (WHO), which was a standard TB treatment comprising the directly observed therapy short course strategy (primary treatment for new cases and primary retreatment for cases of relapse) [11].

Laboratory diagnosis of APTM and DM

The APTB diagnosis was established using the following WHO criteria: sputum smear microscopy with Ziehl-Neelsen staining positive for acid-fast bacilli and/or positive culture for the Mtb complex and/or positive molecular test for Mtb (Xpert MTB/Rif) [34]. The status of DM was established according to the American Diabetes Association, based on fasting blood glucose concentration ≥126 mg/dL or glycated hemoglobin (A1c) values ≥6.5 %. A1c determination was also used to determine the level of glycemic control in patients with DM [35].

Monitoring the response of patients with APTB to standard anti-tuberculosis treatment

TB treatment failure and TB relapse are often accompanied by a delay in sputum smear and culture conversion in patients with APTB [36,37]. For this reason, sputum smear microscopy was performed monthly during treatment. In addition to routine blood analytes, glycemic control was determined by measuring A1c at the beginning of standard six-month TB treatment, halfway through (at three months), and at the end of treatment. Sialic acid, CRP, and cytokines were determined prior to and at the end of the TB treatment.

Blood samples and measurement of routine analytes

For the measurement of routine analytes, fasting (8-10 h) blood samples were obtained on the appointed day by venipuncture, using BD Vacutainer tubes with and without ethylenediaminetetraacetic acid potassium (EDTA-K2) anti-coagulant. An aliquot of whole blood with EDTA-K2 was used for the subsequent determination of A1c. Routine biochemical parameters were determined in serum and the remaining serum and plasma were stored in aliquots of 200 μL at -80°C for later determination of sialic acid, CRP, and cytokines [38-40]. A1c quantification was done on a whole blood aliquot with EDTA-K2 using a latex immunoagglutination inhibition method, and a semiautomatic analyzer (DCA Vantage; Munich, Germany: Siemens). The following analytes were determined in serum using colorimetric and enzymatic methods from Randox (Crumlin, UK: Randox Laboratories) and a semiautomatic clinical chemistry analyzer (Spinlab; Girona, Spain: SPINREACT): glucose, total cholesterol, high-density lipoprotein (HDL) cholesterol, low-density lipoprotein (LDL) cholesterol, triglycerides, total protein, albumin, gamma-glutamyl transferase (GGT), alanine aminotransferase (ALT), aspartate aminotransferase (AST), creatinine, and uric acid. The values of globulins and the albumin/globulin ratio were calculated based on the results of total protein and albumin.

Determination of cytokines

To measure cytokine levels, aliquots of EDTA-K2 plasma stored at -80°C were thawed at 25°C in a single step [38,39]. The cytokine levels were determined according to the manufacturer's instructions. The cytokines measured were IFN-γ (R&D Systems, cat. DIF50C), TNF-α (PeproTech, cat. 9000-K25), IL-6 (PeproTech, cat. 900-K16), IL-10 (PeproTech, cat. 900-K21), IL-8 (PeproTech, cat. 900-K18), and TGF-β1 (R&D Systems, cat. DB100B). All readings were obtained using a Bio-Rad Benchmark Microplate Reader analyzer (Hercules, CA: Bio-Rad Laboratories). The selection of cytokines for evaluation was based on those involved in the inflammatory process, which are observed in both APTB and DM [41-43]. This panel of cytokines comprises proinflammatory types, such as IFN-γ and IL-8, which trigger inflammation, and significant inducers of the acute phase response like TNF-α and IL-6. Additionally, it includes conditionally anti-inflammatory cytokines, such as IL-10 and TGF-β1 [41-43].

Determination of C-reactive protein and sialic acid

For the determination of C-reactive protein, total sialic acid, and free sialic acid aliquots of blood serum stored at -80°C were used after being thawed in a single step at 25°C [38,40]. The quantification of C-reactive protein was carried out by nephelometry following the manufacturer's instructions (MININEPH, cat. ZK500.R) using the MININEPHPLUS analyzer (Birmingham, UK: The Binding Site Group Ltd). Total and free sialic acid were also determined according to the manufacturer's instructions (BioAssay Systems, cat. DSLA-100; Hayward, CA: BioAssay Systems) using the colorimetric Warren method and a spectrophotometer (Spectronic Unicam Genesys 8; Melville, NY: Spectronic Instruments Inc.) [44,45]. The values of total and free sialic acid were used to calculate the values of bound sialic acid [44,45].

Data analysis

The characteristics of subjects with APTB and those with TBDM were described using median and interquartile range (Q1 and Q3) for non-parametric or mean and standard deviation for parametric quantitative variables, and numbers and percentages for qualitative variables. To evaluate the association between two categorical variables, the Pearson chi-square test was used, with p-values simulated by Monte Carlo simulation based on 10,000 repetitions. The quantitative variables were transformed using the Box-Cox method, and comparisons of independent means of transformed variables were performed using the independent samples t-test.

A principal component analysis model was used with the variables IL-8, IL-6, IL-10, TGF-β1, TNF-α, IFN-γ, C-reactive protein, and total sialic acid transformed (using the Box-Cox method) and standardized [46]. This model was used to test whether the change of inflammatory component was different in subjects with TBDM after the six-month standard TB treatment, in comparison to subjects with APTB. Additionally, a vector analysis was performed to observe the correlation between the variables and the correlation with each of the first two principal components of each model. All statistical analysis was conducted using R studio version 4.0.4 (Vienna, Austria: R Foundation for Statistical Computing) [47]. Two-tailed hypothesis tests were used, and the significance level was ≤0.05.

## Results

Baseline characteristics of study subjects

In this study, 30 subjects with APTB (TB relapse: 20%, median age: 33 years, male sex: 67%) and nine subjects with TBDM (TB relapse: 0%, type 2 DM n=8, median age: 47 years, male sex: 56%) were examined (Table 1). All participants displayed characteristic signs and symptoms of APTB, such as a productive cough, hemoptysis, fever, weight loss, asthenia, diaphoresis, headache, hyporexia, weakness, and nausea. Furthermore, no clinical differences were found between the two groups, except for the prevalence of malnutrition, which was higher in subjects with APTB (57%) compared to TBDM subjects (0%), and mean BMI, which was lower in APTB (18 kg/m^2^) compared to TBDM subjects (27.5 kg/m^2^) (Table 1).

**Table 1:** Baseline characteristics of subjects with active pulmonary tuberculosis (APTB) and diabetes mellitus (TBDM) and subjects with active pulmonary tuberculosis without diabetes mellitus. ^*^Cut-off points proposed by the World Health Organization (males and females 20 years and older): underweight <18.5 kg/m^2^, normal 18.5-24.9 kg/m^2^, overweight 25-29.9 kg/m^2^, and obesity ≥30 kg/m^2^. **P-value calculated using Pearson's chi-square test with p-value simulated by Monte Carlo simulation (based on 10000 repetitions). ***The data were transformed using the Box-Cox method and p-value was calculated using an independent samples t-test. A p-value <0.05 was considered significant. Tuberculosis relapse - the reappearance of signs and symptoms in a patient who, having been declared cured or had completed treatment, presents positive bacilloscopy and/or culture again. Data are presented as n (%) unless otherwise indicated. AFB: acid-fast bacilli; BMI: body mass index; IQR: interquartile range (Q1-Q3); TB: tuberculosis

Characteristics		TBDM, n=9	APTB, n=30	p-Value
Sex, n (%)	Male	5 (56)	20 (67)	0.7^**^
	Female	4 (44)	10 (33)	
	Age in years (median, IQR)	47 (35-48)	33 (23.2-51.7)	0.3^***^
	Tuberculosis relapse, n (%)	0 (0)	6 (20)	0.3^**^
	History of TB contact, n (%)	4 (44)	9 (31)	0.7^**^
	Alcoholism, n (%)	3 (33)	9 (31)	1.0^**^
	Smoking, n (%)	2 (22)	14 (47)	0.3^**^
	Consumption of illicit substances, n (%)	1 (11)	14 (47)	0.1^**^
	Malnutrition, n (%)	0 (0)	17 (57)	<0.001^**^
Signs and symptoms	Productive cough, n (%)	9 (100)	29 (97)	1.0^**^
	Hemoptysis, n (%)	4 (44)	8 (27)	0.4^**^
	Fever, n (%)	6 (67)	21 (70)	1.0^**^
	Weight loss, n (%)	4 (44)	22 (73)	0.2^**^
	Asthenia, n (%)	3 (33)	20 (67)	0.1^**^
	Diaphoresis, n (%)	1 (11)	18 (60)	0.02^**^
	Headache, n (%)	2 (22)	10 (33)	0.7^**^
	Hyporexia, n (%)	1 (11)	6 (20)	0.7^**^
	Weakness, n (%)	3 (33)	13 (43)	0.7^**^
	Nausea, n (%)	0 (0)	5 (17)	0.3^**^
	BMI kg/m^2^ (median, IQR)	27.5 (24.8-29.1)	18 (17.4-20.4)	<0.001^***^
Body weight^*^, n (%)	Underweight	0 (0)	15 (50)	<0.001^**^
	Normal weight	3 (33)	12 (40)	
	Overweight	4 (44)	3 (10)	
	Obesity	2 (22)	0 (0)	
Results of sputum smear microscopy	Sputum smear microscopy, n (%)			
	No AFB observed	1 (11)	1 (3)	0.6^**^
	Positive (+)	4 (44)	8 (27)	
	Positive (++)	2 (22)	8 (27)	
	Positive (+++)	2 (22)	13 (43)	
Results of the determination of hemoglobin HbA1c	HbA1c <7%, n (%)	5 (56)	30 (100)	0.001^**^

At the time of their APTB diagnosis, 44% of subjects with TBDM had an A1c greater than 7% (Table 1). However, results of sputum smear microscopy prior to initiating anti-TB therapy showed no significant difference between APTB and TBDM subjects (Table 1).

Total cholesterol, LDL-cholesterol, and triglyceride levels were significantly lower in subjects with APTB compared to those with TBDM (Table 2). Nevertheless, subjects with TBDM did not have hypertriglyceridemia or a significant increase in LDL-cholesterol, considering recommended values [48]. HDL-cholesterol levels were below recommended values (less than 60 mg/dL) in both patient groups [48].

**Table 2:** Baseline biochemical parameters and inflammatory mediators in subjects with active pulmonary tuberculosis (APTB) and diabetes mellitus (TBDM) and subjects with active pulmonary tuberculosis without diabetes mellitus. *The data are shown as median (Q1 and Q3). **The data are shown as mean (SD) (Q1 and Q3). The data were transformed using the Box-Cox method and were analyzed using an independent samples t-test. A p-value <0.05 was considered significant. The following analytes were determined in the serum of nine subjects with TBDM and 30 subjects with APTB: total cholesterol, HDL-cholesterol, LDL-cholesterol, triglycerides, total proteins, albumin, globulins, and albumin/globulin ratio, gamma-glutamyl transferase (GGT), alanine aminotransferase (ALT), aspartate aminotransferase (AST), creatinine, uric acid, C-reactive protein (PCR), free sialic acid, total sialic acid and bound sialic acid. Tumor necrosis factor α (TNF-α), interferon-gamma (IFN-γ), interleukin 6 (IL-6), interleukin 10 (IL-10), interleukin 8 (IL-8), and transforming growth factor β1 (TGF-β1) were determined in plasma. HDL: high-density lipoprotein; LDL: low-density lipoprotein

Parameter	TBDM, n=9	APTB, n=30	p-Value
Total cholesterol (mg/dL)^*^	171.9 (31.6)	144.2 (38.5)	0.044
HDL-cholesterol (mg/dL)^*^	37.2 (10.3)	32.1 (12.3)	0.2
LDL-cholesterol (mg/dL)^**^	105 (92-116)	77 (57.7-99.2)	0.005
Triglycerides (mg/dL)^**^	98 (80-141)	68 (59-88.5)	0.046
Total proteins (g/dL)^*^	6.9(0.5)	7.1 (0.7)	0.4
Albumin (g/dL)^*^	3.7 (0.9)	3.9 (0.7)	0.6
Globulins (g/dL)^*^	3.2 (1.1)	3.2 (0.9)	1.0
Albumin/Globulin ratio^**^	1.3 (0.8-2.1)	1.2 (0.9-1.6)	0.8
GGT (U/L)^**^	27 (20-35)	23 (18.2-40.5)	0.7
ALT (U/L)^**^	20 (14-25)	18.5 (12.2-27.5)	0.9
AST (U/L)^**^	20 (19-23)	23.5 (17-30.7)	0.1
Creatinine (mg/dL)^**^	0.9 (0.6-1.1)	0.8 (0.7-0.9)	0.6
Uric acid (mg/dL)^**^	3.2 (3.1-4.7)	3.3 (1.9-4.7)	0.3
TNF-α (pg/mL)^**^	134 (105.7-296)	229.5 (147.1-522.1)	0.7
IFN-γ (pg/mL)^**^	9.8 (3.4-9.8)	9.8 (8.1-14.3)	0.3
IL-6 (pg/mL)^**^	55.8 (29.6-83.1)	47.6 (35.9-71.6)	0.5
IL-10 (pg/mL)^**^	80.7 (80.7-186.1)	183.7 (45.8-183.7)	0.9
IL-8 (pg/mL)^**^	24.9 (24.9-44.7)	42.7 (27.6-57.9)	0.7
TGF-β1 (pg/mL)^**^	10390 (8609-14800)	2090 (699.4-3716.8)	<0.001
CRP (mg/L)^**^	24.1 (11.9-43.3)	50.2 (24.7-96.2)	0.1
Free sialic acid (mg/dL)^**^	0.8 (0.8-0.9)	1.1 (0.6-1.4)	0.1
Total sialic acid (mg/dL)^*^	89.9 (16.1)	104.1 (28.9)	0.1
Bound sialic acid (mg/dL)^*^	89.1 (16)	103 (28.6)	0.1

The concentrations of acute-phase reactants such as CRP (>8 mg/L) [19,49] and total sialic acid (>2.22 mmol/L ≥68.65 mg/dL) were elevated in both groups, with a trend towards higher values in subjects with APTB (Table 2) [44]. However, no significant differences were found in either marker when both groups were compared. It is noteworthy that TGF-β1 was the only inflammatory mediator that showed a significant difference between groups at baseline, being higher in TBDM subjects (median TGF-β1: 10390 pg/mL) than in APTB subjects (median TGF-β1: 2090 pg/mL) (p<0.001) [29].

Sputum smear microscopy in subjects with good glycemic control after six months of standard TB treatment

Negative results in sputum smear microscopy were found in subjects with APTB and in subjects with TBDM with good glycemic control after six months of standard TB treatment (Table 3). Approximately 56% of subjects with TBDM commenced standard TB treatment with good glycemic control (A1c ≤7%). Additionally, the same percentage of subjects maintained this good glycemic control throughout the treatment period. The median A1c before initiating anti-TB treatment was 6.9% (Q1: 6.7%, Q3: 11.6%), and after six months of anti-TB treatment, it was 6.9% (Q1: 5.9%, Q3: 7.5%). It is likely that due to the good glycemic control, the presence of DM did not impact the results of the sputum smear microscopy at any time after starting anti-TB chemotherapy, as no association (according to the chi-squared test) was found between DM and the results of the sputum smear microscopy [50,51]. This information is important since it provides data on how the negativization of smear microscopy in subjects with TBDM depends largely on glycemic control, an effect that may be related to the reduction of the negative effects that poorly controlled DM has on the interaction of the causative agent of TB with cells of the innate immune system [52,53].

**Table 3:** Results of sputum smear microscopy at baseline and at the completion of a six-month anti-tuberculosis treatment. *Simulated p-value cannot be calculated with zero marginals. **The patient did not attend the follow-up sputum smear microscopy examinations in the fifth and sixth months; the result of the follow-up sputum smear microscopy in the fourth month is shown. (-) No acid-fast bacilli (AFB) were observed in 100 observed fields. (+) 10 to 99 AFB were observed in 100 observed fields. (++) One to 10 AFB per field were observed in 50 observed fields. (+++) More than 10 AFB per field were observed in 20 observed fields. The table represents the comparison of the two groups (TBDM vs. APTB). Sputum smear microscopy was performed monthly during the treatment period. In patients with negative sputum smear microscopy results, tuberculosis diagnosis was confirmed by molecular biology (Xpert MTB/RIF). A p-value of <0.05 was considered significant. APTB: active pulmonary tuberculosis; DM: diabetes mellitus

Sputum smear microscopy (diagnosis and follow-up)	Diagnosis	Six-month follow-up
p-Value	0.562	*
TBDM (n=9)	-	-
	+	-
	+	-
	+	-
	+	-
	++	-
	++	-
	+++	-**
	+++	-
APTB (n=30)	-	-
	+	-
	+	-
	+	-
	+	-
	+	-
	+	-
	+	-
	+	-
	++	-
	++	-
	++	-
	++	-
	++	-
	++	-
	++	-
	++	-
	+++	-
	+++	-
	+++	-
	+++	-
	+++	-
	+++	-
	+++	-
	+++	-
	+++	-
	+++	-
	+++	-
	+++	-
	+++	-

Levels of inflammation in subjects with APTB and concomitant DM

The systemic inflammatory response is characterized by the production of chemokines and cytokines by the cells of the immune system, which participate in and promote multiple biological processes [54]. It has been observed that the concentration of multiple inflammatory mediators, which characterize the systemic inflammatory process, undergoes changes in subjects with active pulmonary tuberculosis who receive anti-tuberculosis treatment, and the results are somewhat controversial [55-62]. In relation to this and considering that both APTB and DM are diseases that cause systemic inflammation, the concurrence of these has been related to a greater burden of systemic inflammation [10,25]. We selected pro-inflammatory and anti-inflammatory cytokines, chemokines, and acute-phase reactants, related to the process of systemic inflammation in both pathologies, to monitor the response to anti-tuberculosis treatment [32].

To estimate systemic inflammation in individuals with TBDM and compare it to those with APTB without DM, the levels of inflammation mediators IL-8, IL-6, IL-10, TGF-β1, TNF-α, IFN-γ, CRP, and total sialic acid were measured prior to standard TB treatment and at the end of it (post-treatment) (Tables 4, 5).

**Table 4:** Values of inflammatory mediators in subjects with active pulmonary tuberculosis with concomitant diabetes mellitus (TBDM) pre- and post-treatment. *The data are shown as median (Q1 and Q3). **The data are shown as mean (SD). The data were transformed using the Box-Cox method and were analyzed using a dependent samples t-test. A p-value <0.05 was considered significant. Values of the following analytes are from the plasma of nine subjects with TBDM: tumor necrosis factor α (TNF-α), interferon-gamma (IFN-γ), interleukin 6 (IL-6), interleukin 10 (IL-10), interleukin 8 (IL-8), and transforming growth factor β1 (TGF-β1). C-reactive protein (PCR), free sialic acid, total sialic acid, and bound sialic acid were determined in serum.

Parameter	TBDM pre-treatment (n=9)	TBDM post-treatment (n=9)	p-Value
IFN-γ (pg/mL)*	9.8 (3.4-9.8)	3.5 (2.9-3.4)	0.012
TNF-α (pg/mL)*	134 (105.7-296)	105.7 (105.7-270)	0.1
IL-6 (pg/mL)*	55.8 (29.6-83.1)	17 (17-42.5)	0.1
IL-10 (pg/mL)*	80.7 (80.7-186.1)	186.1 (5.8-186.1)	0.4
IL-8 (pg/mL)*	24.9 (24.9-44.7)	24.9 (24.9-34.8)	0.4
TGF-β1 (pg/mL)*	10390 (8609-14800)	8422 (6655-11320)	0.5
CRP (mg/L)*	24.1 (11.9-43.3)	5.7 (1.6-9.5)	0.026
Free sialic acid (mg/dL)*	0.8 (0.8-0.9)	0.9 (0.6-1.2)	0.6
Total sialic acid (mg/dL)**	89.9 (16.1)	71.1 (11.4)	0.019
Bound sialic acid (mg/dL)**	89.1 (16)	70.2 (11.3)	0.018

**Table 5:** Values of inflammatory mediators in subjects with active pulmonary tuberculosis without diabetes mellitus (APTB) pre- and post-treatment. *The data are shown as median (Q1 and Q3). **The data are shown as mean (SD). The data were transformed using the Box-Cox method and were analyzed using a dependent samples t-test. A p-value <0.05 was considered significant. The following analytes were determined in plasma of 30 subjects with APTB: tumor necrosis factor α (TNF-α), interferon-gamma (IFN-γ), interleukin 6 (IL-6), interleukin 10 (IL-10), interleukin 8 (IL-8) and transforming growth factor β1 (TGF-β1). C-reactive protein (PCR), free sialic acid, total sialic acid, and bound sialic acid were determined in serum.

Parameter	APTB pre-treatment (n=30)	APTB post-treatment (n=30)	p-Value
IFN-γ (pg/mL)*	9.8 (8.1-14.3)	3.4 (2.9-5.2)	<0.001
TNF-α (pg/mL)*	229.5 (147.1-522.1)	144.8 (106.1-233.5)	0.2
IL-6 (pg/mL)*	47.6 (35.9-71.6)	13.4 (3.9-35.9)	<0.001
IL-10 (pg/mL)*	183.7 (45.8-183.7)	27.6 (27.6-100.4)	0.019
IL-8 (pg/mL)*	42.7 (27.6-57.9)	42.7 (25.4-64.4)	0.6
TGF-β1 (pg/mL)*	2090 (699.4-3716.8)	4779 (2275-8666)	0.013
CRP (mg/L)*	50.2 (24.7-96.2)	3.9 (1-9.4)	<0.001
Free sialic acid (mg/dL)*	1.1 (0.6-1.4)	0.8 (0.6-0.9)	0.1
Total sialic acid (mg/dL)**	104.1 (28.9)	68.1 (12.8)	<0.001
Bound sialic acid (mg/dL)**	103 (28.6)	67.3 (12.85)	<0.001

**Figure 1:**
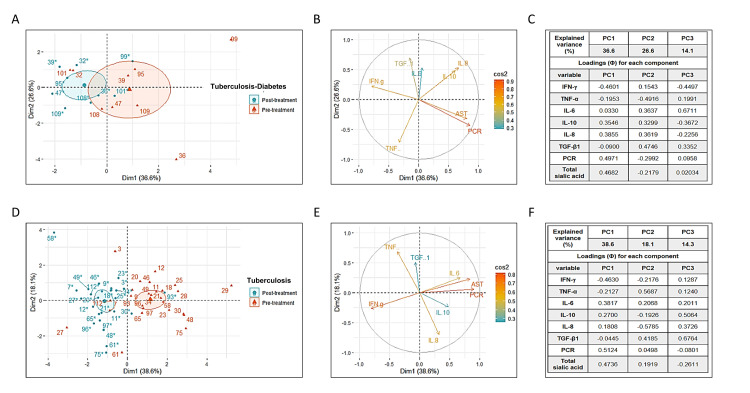
Evidence of persistent inflammation in subjects with TBDM and evidence of change in inflammatory response in subjects with APTB after six months of standard tuberculosis treatment. Inflammatory mediators were measured in samples from APTB and TBDM patients before and after anti-tuberculosis treatment. (A) Using principal component analysis (PCA1) with selected inflammatory mediators (IL-8, IL-6, IL-10, TGF-β1, TNF-α, IFN-γ, C-reactive protein, and total sialic acid), initial responses of TBDM subjects could not be distinguished from treatment responses. First and second principal components (PC1 and PC2) are displayed. (B) Vector analysis revealed positive correlations between total sialic acid and CRP, and a negative correlation with IFN-γ. (C) Total sialic acid, CRP, and IFN-γ showed the highest loadings in PCA1. (D) PCA2, based on inflammatory mediators, distinguished pre- and post-treatment responses in APTB patients. (E) Positive correlations were found between total sialic acid and CRP, and a negative correlation with IFN-γ in PCA2. (F) Total sialic acid, CRP, and IFN-γ had the highest loadings, aiding in distinguishing pre- and post-treatment responses in the APTB group. DM: diabetes mellitus; APTB: active pulmonary tuberculosis

Principal component analysis (PCA) was used to explore the values of these mediators and avoid redundant information (Figures 1A-1F) [63]. The systemic inflammatory component, explained by the determined inflammatory mediators, showed a persistence of inflammation in TBDM subjects. A PCA model (PCA1) was obtained with the pre- and post-treatment values of the eight inflammatory mediators. By considering the first three principal components (PC1, PC2, and PC3), the PCA1 model explained 77.3% of the total variability. The results showed no discernible differences between pre- and post-treatment inflammatory responses (Figures 1A-1C).

For the group with APTB, the PCA model 2 (PCA2) explained 71% of the total variability. The first three principal components (PC1, PC2, and PC3) helped distinguish between pre- and post-treatment inflammatory responses (Figure 1D). Further analysis using vector and load analyses showed that IFN-γ, CRP, and total sialic acid (Figure 1E) were the markers that best helped distinguish between the pre- and post-treatment responses of the APTB group (load Φ: PC1 IFN-γ = - 0.4630, CRP = 0.5124, total sialic acid = 0.4736) (Figure 1F).

## Discussion

The present study aimed to assess the changes that occur in inflammatory markers of individuals with APTB and TBDM from baseline and the end of the six-month standard TB treatment. Our findings revealed that both groups of subjects displayed negative results in the control test of sputum smear microscopy after six months of anti-TB chemotherapy. Subjects with TBDM showed good glycemic control alongside the negative results of the sputum smear microscopy after the same period. However, systemic inflammation, as explained by various inflammatory mediators, including IL-8, IL-6, IL-10, TGF-β1, TNF-α, IFN-γ, CRP, and total sialic acid, persisted post-treatment in individuals with TBDM but not in those with APTB. Additionally, IFN-γ, CRP, and total sialic acid were the inflammation mediators that had the most significant impact on distinguishing between the pre- and post-treatment inflammatory response in individuals with APTB.

All subjects in the study experienced symptoms and signs typical of APTB, and no significant baseline clinical differences were found between individuals with APTB and those with TBDM. This finding may apparently contradict other studies that have reported TBDM comorbidity to be associated with greater TB severity and a more severe clinical presentation of TB in individuals with TBDM compared to those with APTB alone [64,65]. However, we consider that this can be explained by the absence of relapse TB cases in the TBDM group, which generally predisposes patients to greater lung injury [66]. The fact that approximately 56% of individuals with TBDM had a good glycemic control at the start of standard TB treatment (A1c <7%), influenced mycobacterial load, improved metabolism, and enhanced immunity against the causative agent of TB in individuals with TBDM [67-69].

The comorbidity of APTB and DM has been found to be associated with prolonged time for sputum smear microscopy and culture conversion, as well as an increased likelihood of treatment failure [70]. In our study, we observed timely negative results of sputum smear microscopy in both APTB and TBDM subjects. Both groups showed negative results of sputum smear microscopy after six months of anti-TB chemotherapy. Factors such as good adherence to anti-TB treatment in both APTB and TBDM subjects, as well as the presence of good glycemic control (A1c <7%) before and during anti-TB chemotherapy in TBDM subjects, could explain these good conversions of sputum smear microscopy [51,67,71].

Patients with APTB had lower levels of total cholesterol, LDL-cholesterol, and triglycerides compared to those with TBDM [7,72]. In addition, HDL-cholesterol levels were below recommended values in both patient groups [7]. It has been described that subjects with TBDM exhibit a phenotype with metabolic characteristics of both DM (such as dyslipidemia) and TB (such as emaciation) [71]. Thus, some TBDM patients may have TB effects that dominate over those of DM, while others may have the opposite phenotype, with DM effects dominating over those caused by TB. The evolution of DM is an important factor in this regard [72,73]. Metabolic characteristics, such as the presence of a hyperglycemic and dyslipidemic environment, can affect the immune system response by interfering with the levels of inflammatory mediators and the development of T cell immunity [74-76]. Thus, metabolic alterations observed in subjects with TBDM (who had different DM evolution) may affect the inflammatory response during anti-TB chemotherapy.

In some individuals with weakened immune systems, there is a higher risk of developing active TB due to a dysregulation of cytokine levels that results in an altered immune response [77]. Our study found that TGF-β1, a cytokine produced by multiple leukocyte lineages, including activated macrophages and regulatory T cells, had higher concentrations in subjects with TBDM than in those with APTB. Active TGF-β1 has been shown to inhibit T cell proliferation, the destruction of infected macrophages, and the destruction of extracellular cells by macrophages [78]. High concentrations of TGF-β1, in the presence of high glucose levels and the absence of IL-6, can also suppress the Th1 response, which is essential during the immune response to Mtb infection [79]. Thus, elevated TGF-β1 levels in subjects with TBDM may affect the immune response against the causal agent and bacterial elimination in granulomas and sterilization of lesions caused by TB [79,80].

Multidimensional analyses revealed that it is not possible to distinguish between pre- and post-treatment inflammatory responses for subjects with TBDM. This persistent inflammation was observed despite negative sputum microscopy results after six months of anti-TB chemotherapy. However, for subjects with APTB, it is possible to distinguish between pre- and post-treatment inflammatory responses. These results suggest that there is a distinct evolution of the inflammatory process in subjects with APTB compared to those with TBDM when receiving the standard anti-TB treatment. This is due to the possible influence of DM on the immune response to TB and its effects on the different elements of the cytokine network during the immune response, as mentioned above [80,81].

The persistence of inflammation (post-treatment) in subjects with TBDM has been previously reported, suggesting that it could signify a delay in the sterilization process of lesions [29]. The differences we observed in APTB subjects, with and without DM, may be indicative that comorbidity leads to a greater pulmonary involvement related to persistent inflammation [3,7,82]. In addition, the differentiation shown by the panel of inflammation markers between APTB patients who completed treatment and their initial state, unlike those with TBDM, suggests a persistent chronic low-grade inflammation in individuals managing TBDM, despite maintaining good glycemic control. Probably, the chronic inflammation observed in our TBDM patients is a consequence of the long-term metabolic dysregulation that these patients experience due to DM [83,84]. Furthermore, the perpetuation of chronic inflammation in TBDM patients could increase the risk of TB relapse in these individuals [28,85]. Therefore, novel approaches are required to monitor these patients.

A key observation of the present study is that the markers IFN-γ, CRP, and total sialic acid are particularly sensitive in distinguishing the inflammatory response in APTB subject’s pre-treatment, compared to that presented in the post-treatment phase. Given this, these three markers could be useful for monitoring subjects with APTB during standard TB treatment. Our findings align with previous studies, which emphasize the significance of monitoring circulating acute phase reactants and cytokines as parameters that aid in distinguishing individuals with TB both before and after standard TB treatment [22,62,86,87]. Thus, the importance of the study lies in the fact that systemic inflammation can be evaluated with the determination of these inflammatory mediators (IFN-γ, CRP, and total sialic acid), and this evaluation could be useful in conjunction with sputum smear microscopy to evaluate the response to anti-tuberculosis treatment in subjects with APTB and TBDM [7,17,29,82].

Our study has various strengths. We analyzed data from patients in Sonora, Mexico, a region with a high prevalence of TB [88]. In this state in the northwest of Mexico, an average of 857 new cases of active pulmonary tuberculosis occurred between 2009 and 2019; however, the incidence in 2019 (37.3 cases per 100000 habitants) doubled the national average (15.6 cases per 100000 habitants) [89]. Furthermore, in Sonora, Mexico, tuberculosis causes around 100 deaths annually [90]. This makes our findings comparable to similar high-prevalence areas in Mexico and elsewhere. Also, we excluded TB laboratory-confirmed HIV coinfection and other self-reported pathologies that could potentially alter the levels of the determined inflammatory mediators. In this way, the risk of alterations in the levels of the determined inflammatory mediators being related to pathologies other than TB or DM was reduced. Additionally, cytokine determinations were performed in EDTA-K2 plasma, as it has been shown that cytokine levels in plasma are more stable than in serum [91].

Limitations

Despite having broad access to comprehensive information from a standardized public health program focused on tuberculosis, which provides clinical data from all medical units in the capital of Sonora, Mexico, there is a possibility that the program has omitted non-target patients, leading to potential selection bias in our study. Furthermore, the study was limited by the small number of subjects included. This limitation arose because the study was conducted during the COVID-19 pandemic period, which precluded the inclusion of all APTB and TBDM patients. In this prospective longitudinal study, the sample size for this cohort was sufficient to compare the characteristics or parameters evaluated, but there is not enough statistical power to perform regression analysis with confounding variables included, so the data presented here serves an exploratory purpose [92,93]. Additionally, it is plausible that the COVID-19 pandemic has had adverse effects on TB and DM management, as some patients may have neglected to visit health centers for treatment during this time [94,95].

## Conclusions

In the present study, we found that inflammatory mediators, especially IFN-γ, CRP, and total sialic acid measured collectively, may be useful for evaluating the systemic inflammatory response in subjects with APTB and TBDM before and after anti-tuberculosis treatment. The determination of these inflammatory mediators allowed us to discover that systemic inflammation persists in subjects with TBDM after six months of standard treatment for tuberculosis, despite presenting negative results in sputum smear microscopy after six months of treatment and good glycemic control. This finding could indicate the need for therapeutic methods that modulate inflammation in these patients during tuberculosis control with anti-tuberculosis treatment. Furthermore, our findings indicate that monitoring the results of sputum smear microscopy together with the determination of the proposed inflammatory mediators (IFN-γ, CRP, and total sialic) is valuable for evaluating the response to anti-tuberculosis treatment of subjects with APTB without DM, a possibility that deserves future investigations.
